# Molecular Dynamics Investigation of *gluazo*, a Photo-Switchable Ligand for the Glutamate Receptor GluK2

**DOI:** 10.1371/journal.pone.0135399

**Published:** 2015-08-26

**Authors:** Yanan Guo, Tino Wolter, Tomáš Kubař, Martin Sumser, Dirk Trauner, Marcus Elstner

**Affiliations:** 1 Department of Theoretical Chemical Biology, Institute of Physical Chemistry, Karlsruhe Institute of Technology, Kaiserstr. 12, 76131, Karlsruhe, Germany; 2 Department of Chemistry, Ludwig-Maximilians-Universität München and Center of Integrated Protein Science, Butenandtstr. 5–13, 81377 Munich, Germany; German Cancer Research Center, GERMANY

## Abstract

Photochromic ligands (PCLs), defined as photoswitchable molecules that are able to endow native receptors with a sensitivity towards light, have become a promising photopharmacological tool for various applications in biology. In general, PCLs consist of a ligand of the target receptor covalently linked to an azobenzene, which can be reversibly switched between two configurations upon light illumination. *Gluazo*, as a PCL that targets excitatory amino acid receptors, in its dark-adapted *trans* iso-form was characterized to be a partial agonist of the kainate glutamate receptor GluK2. Application of UV light leads to the formation of the *cis* form, with remarkedly reduced affinity towards GluK2. The mechanism of the change of ligand affinity induced by the photoisomerization was unresolved. The presented computational study explains how the isomerization of such a PCL affects the structural changes in the target receptor that lead to its activation.

## Introduction

Glutamate is the most important excitatory neurotransmitter in normal brain function. The synaptic actions of glutamate are regulated by two distinct classes of glutamate receptors predominantly distributed on central neuronal synapses: metabotropic and ionotropic glutamate receptors. The former are G-protein coupled receptors (mGluRs), and the latter are ligand-gated cationic channels (iGluRs), which are essential for the fast synaptic transmission between neurons. Three subfamilies of iGluRs have been identified, and are named after their specific agonists: *α*-amino-3-hydroxy-5-methyl-4-isoxazole-propionic acid receptors (AMPARs), kainate receptors (KARs), and N-methyl-D-aspartate receptors (NMDARs) [1, 2]. All of the iGluRs are nonselective cation channels, allowing the passage of K^+^ and Na^+^, and NMDARs are permeable to Ca^2+^. The three iGluR subfamilies perform very distinct functions at the synapse and neural processing. KARs differentiate themselves functionally as unconventional members of the iGluR family [3, 4]. They play key roles in both presynaptic and postsynaptic neurons. Presynaptic KARs modulate both excitatory and inhibitory neurotransmitter release; postsynaptic KARs mediate excitatory neurotransmission [5–7]. These varied roles generally affect the balance between the excitation and the inhibition in neuronal networks, thereby influencing oscillatory behavior and, potentially, cognition [8, 9].

Structurally, just like AMPARs and NMDARs, KARs are typically tetrameric proteins composed of various combinations of five types of subunits, GluK1 through GluK5. Each of the subunits has a modular structure, consisting of the extracellular N-terminal domain (NTD), the extracellular ligand-binding domain (LBD), the channel-forming transmembrane domain (TMD), and the intracellular C-terminal domain (CTD) [2]. In the LBD, two subdomains (upper lobe, D1 and lower lobe, D2) form a clamshell-like arrangement. The LBD features a binding site for ligands, which can be agonists or antagonists. A number of GluK2 LBDs with bound ligands have been resolved by X-ray crystallography [10, 11], providing insight into the movement of the receptor and the selectivity of ligand in the receptor gating [12, 13].

The Trauner lab has recently introduced the concept of photopharmacology and, more specifically, the application of photochromic ligands (PCLs) to endow native receptors with sensitivity towards light. Among many successfully targeted receptors, PCLs acting on iGluRs were developed [14, 15]. PCLs can be switched reversibly between two conformations (*cis/trans*) with light, thereby altering their affinity towards their target receptor. In general, these molecules contain a light-sensitive azobenzene group attached to the respective ligand.

**Fig 1:**
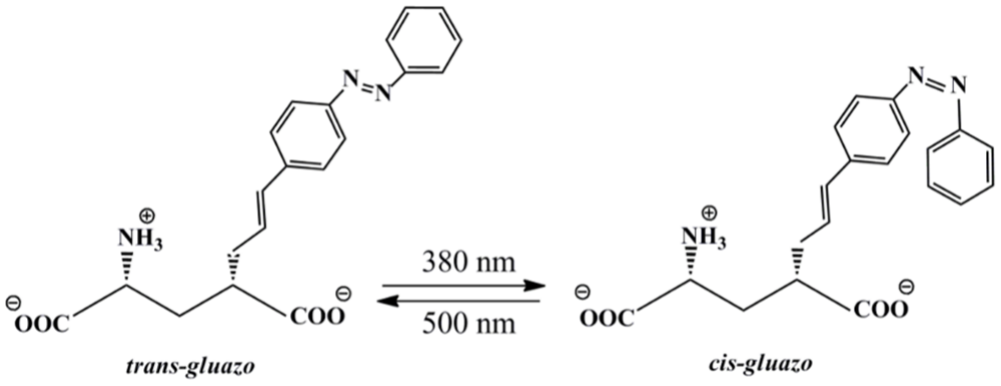
The photo-isomerization of zwitterionic *gluazo*.

In a previous work, we modeled the effect of the PCL ATA-3 on AMPA receptors [16]. Now, given the availability of a crystal structure of *gluazo* bound to the LBD of its target receptor GluK2 [17], it is the first time that we can simulate the mechanism of structural changes in the target receptor induced by the isomerization of such a PCL, leading to the activation of the receptor. Following illumination with UV light, the structure of *gluazo* changes from an extended and flat in the *trans* configuration to a bulky geometry in the cis configuration, see Fig 1. Following on our previous studies of GluA2 [16, 18], we have employed molecular dynamics, an ideal tool for investigation of the structure and dynamics of biomolecular systems, to probe the mechanism of response of GluK2 to ligand photoisomerization.

## Methods

The starting point for the current study is the structural model of the complex of *trans-gluazo* bound to the GluK2 LBD monomer (referred to as GluK2-*trans* further), PDB ID 4H8I [17]. The amino-acid residues that had not been resolved in the experiment were added using Modeller v9.13; these were single residues at the termini of the peptide chain. The complete structure of the GluK2-*trans* complex is shown in S1 Fig. The complex was embedded in a periodic rectangular box sized 7.6 nm × 7.1 nm × 6.8 nm, solvated with ca. 10,000 TIP3P [19] water molecules and neutralized with sodium cations [20]. Amber99SB force field [21] was used to describe the complex, while the ligand was modelled with the general amber force field (GAFF) [22]. The atomic charges of the ligand were obtained with the RESP procedure [23], using Gaussian03 [24] and Antechamber (component of AmberTools) [25] for the calculation.

An equilibration procedure was performed prior to the production simulations. It started with a steepest descent minimization of 2000 steps, followed by the generation of initial velocities on the basis of Maxwell–Boltzmann distribution at 300 K, and a two-phase MD equilibration with position restraints applied to the heavy atoms of GluK2-*gluazo*. The first phase consisted of a simulation of 500 ps with temperature maintained at 300 K using the Nosé–Hoover thermostat [26, 27]. Additionally, the second phase of 500 ps involved an isotropic Parrinello–Rahman barostat [28] to maintain the pressure at 1 bar. Then, the force constant of the harmonic restraints imposed on the solute was reduced gradually from 1000 to 100 kJ⋅mol^−1^⋅nm^−2^ in 10 ns, and then to 10 kJ⋅mol^−1^⋅nm^−2^ in 20 ns before the restraints were abandoned altogether. As for the production runs, five independent simulations of 500 ns with different initial velocities were conducted. The simulations employed a time step of 2 fs, and the Lincs algorithm was applied to keep all of the covalent bonds constrained at their respective reference distances. Regarding the treatment of non-bonded interactions, a cut-off of 1.0 nm was used for the van der Waals interactions while the point-charge electrostatics were evaluated with the particle–mesh Ewald implementation [29]. All of the MD simulations were performed with the GROMACS package, version 4.6.1 [30, 31].

Following our previous work [16], MD simulations involved an LBD monomer rather than a dimer or even higher oligomer. One reason for this choice is to save computational resources because already simulations of LBD monomer are costly. The other is to avoid any sampling issues stemming from the extremely weak motions of the monomers relative to each other. The choice of simulating a monomer was assessed in Ref. [16] (supporting information). Importantly, the internal dynamics of the LBD was shown to be the same in the monomer and in the dimer. There were differences, also: (i) The clamshell was able to open wider in a free monomer due to the lack of inter-subunit contacts, which restricted the clamshell opening in an LBD dimer. (ii) The response of the channel (or the linker replacing the channel) induced by the opening of the clamshell was smaller in an LBD dimer than in the monomer, by virtue of the interfering dynamics of the interface between monomers. Any consequences of these issues will be discussed where applicable in the current work. Notably, the most important qualitative results—the identification of interactions governing the ligand binding and the characterization of protein response to the ligand isomerization—will be unaffected.

## Results

### Structure of the complex with *trans-gluazo*

The mode of *gluazo* binding to the receptor that occurs in the available X-ray structure may not be the only one possible, taking into account the considerable physical size of the ligand. Therefore, several independent simulated annealing simulations at temperatures of up to 450 K were used to search for a possible different ligand binding mode, and one such mode was found indeed. However, additional free energy calculations suggested a more favorable, lower free energy for the ligand binding mode that is present in the X-ray structure than for that in the newly found structure. Therefore, the present work focuses on the X-ray binding mode. The details of the newly found structure may be referred to S1 Text.

### Isomerization of *gluazo*

A forced switching simulation was performed on the GluK2-*trans* complex to investigate the receptor response to the *trans-cis* photo isomerization of *gluazo*. The model of photo-induced dynamics used here was initially proposed by Nguyen et al. [32], and was applied in previous research in our laboratory successfully [16]. Thus, a biasing potential of the form $V_{\text{add}}=\frac{1}{2}K(1+\text{cos CNNC})$ with *K* = 320 kJ⋅mol^−1^ was imposed to force the rotation of 180° along the N = N bond (the dihedral angle CNNC), completing the isomerization from *trans* to *cis*. The biasing potential was discarded after 500 fs, in each of the simulations.

The final structures from the extended equilibration procedure were used as the initial conditions for the switching simulation. The CNNC dihedral angle turned from 180° to ca. 0° rapidly, accomplishing the isomerization within 100 fs, which is in accordance with the previous simulation reports of the isomerization occurring within 250 fs [32–35]. The CNNC dihedrals after 100 fs was −3.0°, 7.8°, −4.2°, 7.7°, and −5.4°, respectively, in the five independent trajectories, and it fluctuated around 0° afterwards (see S2 Fig).

To see if the isomerization affects the structure of the receptor on this short time scale, the root mean square deviation (RMSD) of the receptor backbone from the starting structure was calculated (see S3 Fig). In all of the five simulations, very small RMSD of ca. 0.02 nm was observed during the first 100 fs. This indicates that the isomerization of *gluazo* does not lead to any immediate structural response of the protein. The same result was observed in our previous study on GluA2 [16]. RMSD increases further to ca. 0.04 nm after 500 fs, which is a value that would be expected even in a free MD simulation. This indicates that while the structure of the binding pocket and its closest vicinity adjusts to the switching of *gluazo* rapidly, the overall protein structure remains rather intact on the sub-picosecond time scale. Any large-scale conformational transitions, which lead to the opening of the channel eventually, will take place more slowly, on a time scale reaching hundreds of nanoseconds possibly.

These switching simulations provided us with structural model of a complex of *cis-gluazo* bound to GluK2, which will be used and referred to as GluK2-*cis* in the following.

### Response of protein to the configurational transition of *gluazo*

**Fig 2:**
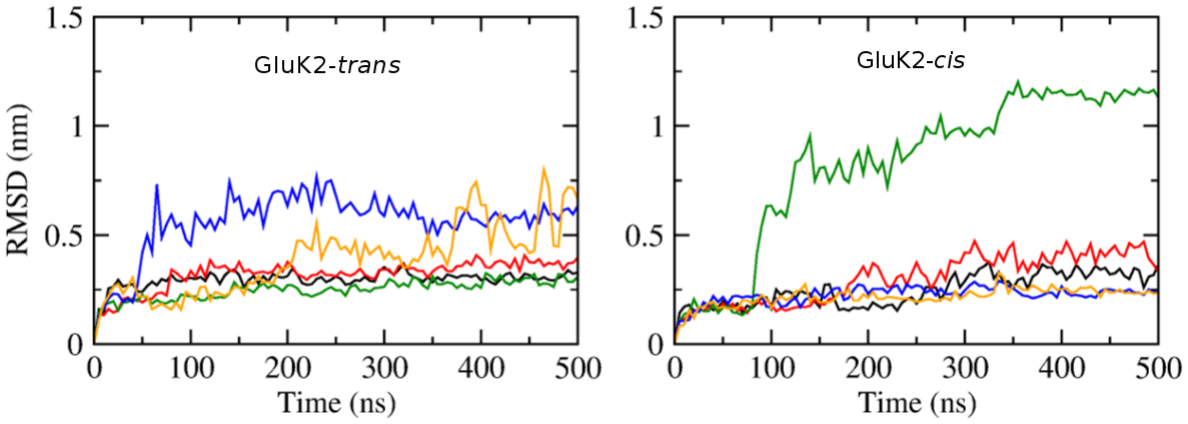
RMSD of the backbone atoms of the receptor with respect to the starting structure in the course of MD simulations. Left: GluK2-*trans* complex; right: GluK2-*cis* complex. Data obtained from five independent simulations of 500 ns each, for each of the complexes. The black, red, green, blue, orange curves correspond to the simulations #1, #2, #3, #4, #5, respectively.

To assess the effect of photo-isomerization on the structure and dynamics of the receptor, five independent MD simulations of 500 ns were performed both on GluK2-*trans* and GluK2-*cis* complexes. Prior to this extended simulation, the GluK2-*cis* complex was subjected to the same equilibration scheme as that for GluK2-*trans*. The RMSD of protein backbone with respect to the starting structure along each trajectory is shown in Fig 2 for both complexes. The value increases gradually to 0.20–0.25 nm at 50 ns in all of the simulations. In the further course of several simulations, a large increase of the RMSD values was observed; an additional analysis showed that this corresponded to the LBD clamshell opening wide (see also below). This may have been a consequence of the simulation setup involving a LBD monomer. Let us recall that such a wide opening of the clamshell is not possible in a LBD oligomer due to sterical clashes of the D2 domains of the different monomers. Thus the wide-open conformation may be over-stabilized in our simulations artificially, while the closed and moderately open (as with a partial agonist) conformations are described correctly. Therefore, in the following discussions, we will not consider the simulations where wide-open conformations occurred, and we will concentrate on the closed and moderately open conformations.

**Fig 3:**
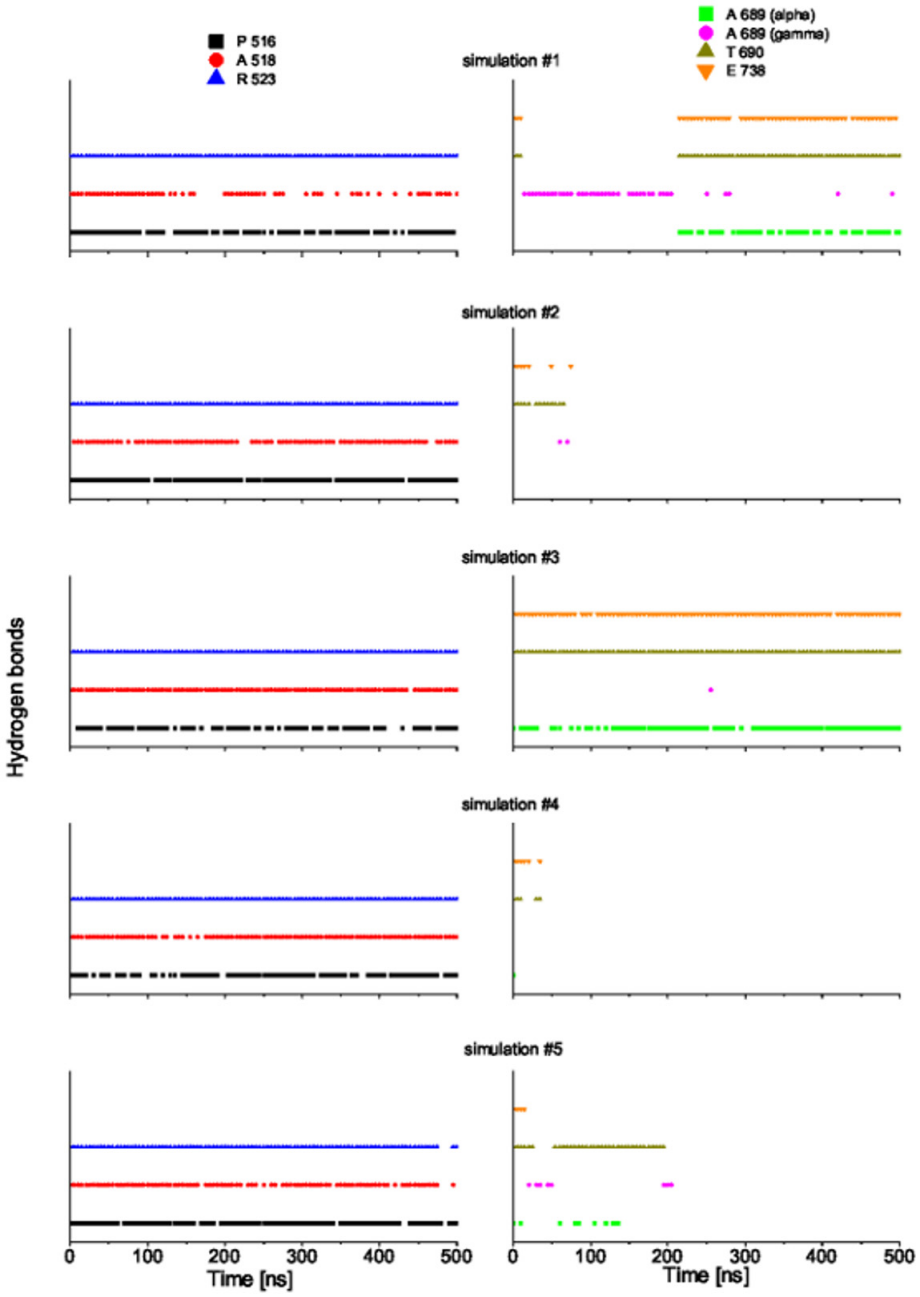
Hydrogen bond patterns in the GluK2-*trans* complex in course of MD simulations of 500 ns. P516: HBs between the *α*-amino acid group of *gluazo* and the backbone carbonyl group of P516; A518: HBs between the *α*-carboxyl group of *gluazo* and the backbone NH group of A518; R523: HBs between the *α*-carboxyl group of *gluazo* and the guanidinium group of R523; A689 (alpha): HBs between the *α*-carboxyl group of *gluazo* and the backbone NH group of A689; A689 (gamma): HBs between the *γ*-carboxyl group of *gluazo* and the backbone NH group of A689; T690: HBs between the *γ*-carboxyl group of *gluazo* and the backbone NH group or the side chain OH group of T690; E738: HBs between the *α*-amino acid group of *gluazo* and the side chain carboxyl group of E738. Data from the simulations #1, #2, #3, #4, #5 from top to bottom.

Among the simulations of GluK2-*trans*, two trajectories show a distinct D1–D2 clamshell opening accompanied by a steep rise of RMSD (simulations #4 and #5, blue and orange in Fig 2). The time course of ligand–protein hydrogen bond (HB) patterns is shown in Fig 3 for each trajectory. The ligand–D1 interactions are preserved even after the clamshell has opened (at ca. 50 ns in #4; at ca. 200 ns in #5). On the other hand, the ligand–D2 interaction is lost upon the opening of the clamshell. While these trajectories probably yield a too-wide open clamshell, this observation supports the argument that the ligand mainly binds to the D1 domain in the open state of LBD clamshell. This is consistent with the previous reports that D1 remains relatively fixed during the opening and closing motion of the clamshell due to the restriction from the D1–D1 interface in the intact receptor [2]. Notably, early spectroscopic work suggested that studies of the LBD clamshell motion should focus on the ligand–D2 interactions because these are involved in the “locking” step of the ligand binding, which presumably corresponds to the clamshell closure.[36, 37]

Two simulations of GluK2-*trans* with comparable RMSD values (trajectories #1 and #2; black and red in Fig 2) show quite different protein stability. That is, the ‘red’ simulation shows the clamshell opening, while the ‘black’ one does not. This suggests that RMSD may not always be a good measure of the conformational change of the protein (especially considering that the protein features flexible loops). In GluA2, the center-of-mass (COM) distance between residues G451 (corresponding to G489 in GluK2) and S652 (D687 in GluK2) had been proposed as a good descriptor of the closing degree of the LBD clamshell [16, 18]. In the present work, the COM distance of residues Y488 and G688 is considered to represent the clamshell opening and closing movement, motivated by the large distance of these residues from the hinge of the clamshell. Also, it can be inferred from the structure of the complex (S1 Fig) that Y488 and G688 are located near the termini of loops connecting secondary structure elements, which will reduce the bias caused by the inherent flexibility of the loops.

**Fig 4:**
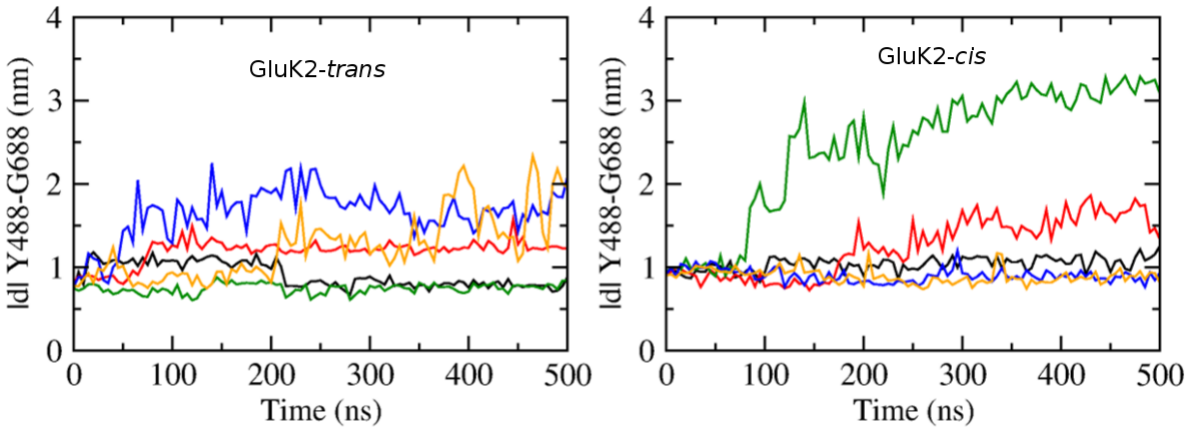
The opening extent of the LBD clamshell in the course of MD simulations, described with the center-of-mass distance of Y488 and G688. Left: GluK2-*trans* complex; right: GluK2-*cis* complex. Data obtained from five independent simulations of 500 ns each, for each of the complexes. The black, red, green, blue, orange curves correspond to the simulations #1, #2, #3, #4, #5, respectively.

The Y488–G688 distance is 0.63 nm in the closed state of GluK2 (PDB ID 1S50, bound with glutamate), and it is 0.82 nm in the partially closed state (PDB ID 2XXT, bound with kainate). Since no X-ray structure of the GluK2 *apo*-protein or a GluK2-antagonist complex is available, we consider the complex of GluK1 bound with the antagonist UBP-315 (PDB ID 2QS1) as the reference for the open state, which features a Y505–G704 COM distance of 1.15 nm (Y505 and G704 correspond to Y488 and G688 in GluK2, respectively). The COM distance of Y488–G688 shown in Fig 4 captures the clamshell opening and closing in GluK2-*trans* well, with the average values of 0.97 nm, 1.19 nm, 0.75 nm, 1.65 nm and 1.25 nm for the five respective simulations #1 through #5. We infer that the simulations #1 and #3 provided a reasonable description of the bound state (moderately open), while the remaining three trajectories suffered from an artificially over-stabilized open state of the clamshell. In most of the following structural considerations, we will concentrate on the trajectories #1 and #3.

For GluK2-*cis*, the protein structure collapses in the simulation #3 after 100 ns (green curve in Fig 4 right, with a large Y488–G688 distance above 2.5 nm). In the simulation #2 (red curve in that figure), *cis-gluazo* dissociates after 260 ns, accompanied by the opening of the clamshell and an increase of the Y488–G688 distance to more than 1.5 nm. The three remaining simulations #1, #4 and #5 show average Y488–G688 distances of 1.04 nm, 0.90 nm and 0.89 nm, respectively. Disregarding the simulations with the wide-open clamshell, we find that both the *trans-gluazo*-bound state and the *cis-gluazo*-bound state show comparable LBD clamshell conformations, corresponding to a partial agonist-bound state in terms of the clamshell opening extent.

### Reaction of the Linker Segments in the Dimer

**Fig 5:**
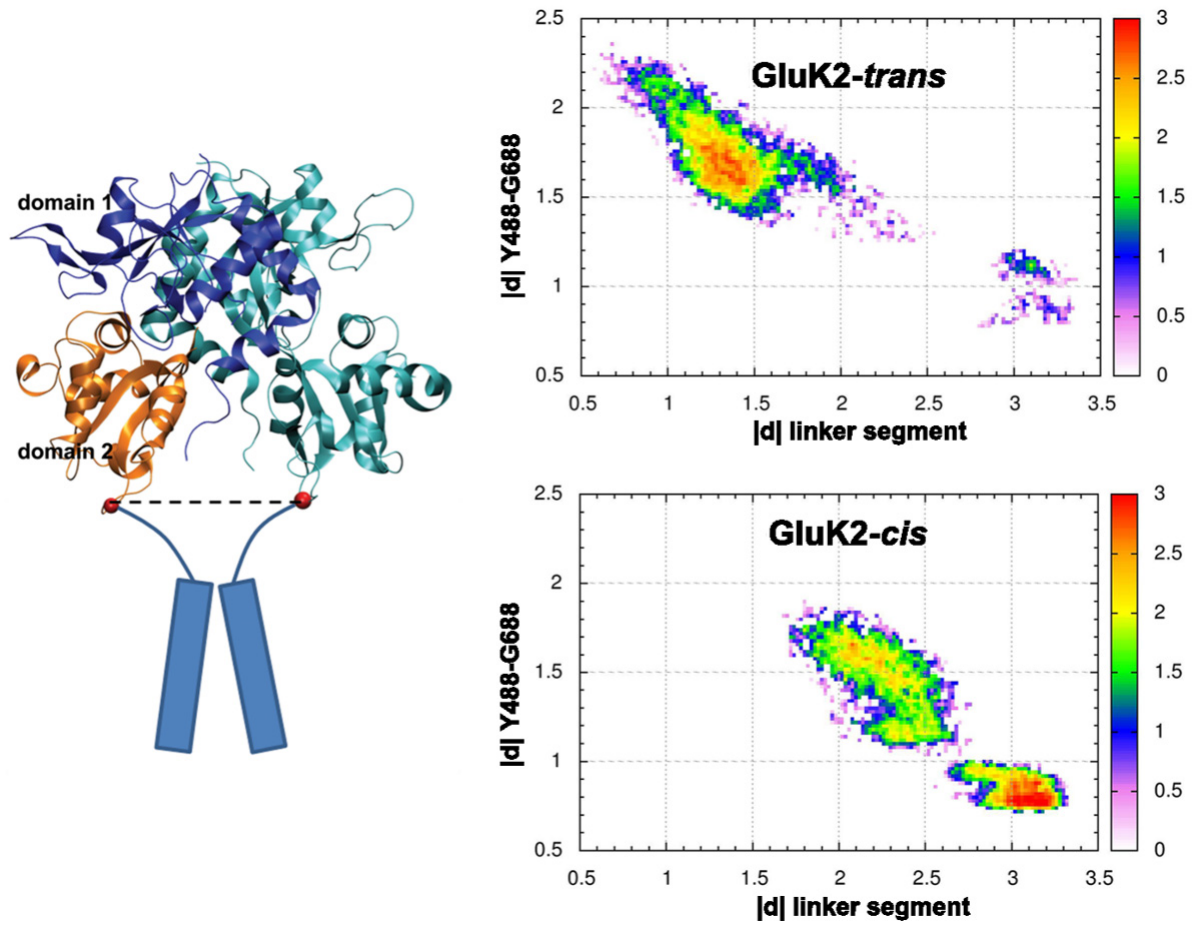
Impact of the conformational changes of the clamshell on TMD. Left: structure of the GluK2 dimer. Blue—D1 domain of the template monomer, orange—D2 domain of the template monomer, cyan—the dynamic monomer, red spheres—centers of mass of the residues at the ends of the linker segment (K544 and P667) that replaces the transmembrane domains (cylinders) in the full-length receptor. Right: correlation of the distance of linkers in monomer 1 and monomer 2 (nm) and the Y488–G688 COM distance (nm), for the complexes GluK2-*trans* and GluK2-*cis*. Probability density coded by color (logarithmic scale, arb. u.). Note that the data were obtained from one MD simulation each and thus cannot reproduce the equilibrium probabilities due to insufficient sampling; the purpose of the plot is merely to illustrate the good linear correlation between the two quantities on a broad interval of clamshell opening extent.

Our MD simulations were performed on monomeric chains of the LBD domain. In order to extrapolate the results to the ion channel reaction, virtual MD trajectories involving a dimer of the receptor were constructed. In this procedure, one monomer from the X-ray structure (PDB ID 4H8I) was used as a template, and the MD trajectory was aligned to the D1 domain of the template monomer. Together with the other monomer in the X-ray structure, this procedure yielded a virtual dimer composed of one dynamic monomer and one static monomer. This made it possible to describe, albeit crudely, the orientation of TMDs. In the LBD crystal structure as well as in our simulations however, the TMD (residues 545 through 666) is replaced by a linker segment GT located at the N-terminus of helix E and connecting the residues K544 and P667; K544 is linked to the transmembrane helix M1, and P667 is linked to the helix M3 in the full-length receptor. Upon the binding of an agonist, the distance between these linker segments increases, and this results in a reorientation of TMD helices and opening of the ion channel (see Fig 5). Therefore, the COM distance of the linkers in monomer 1 (in the simulation, aligned to the template) and in monomer 2 (stationary) is regarded as a proxy of the opening and closing of the ion channel [38].

To measure the distance between the linkers, each linker was represented by the COM of the four-residue segment K544-G-T-P667. The correlation of the linker distance and the Y488–G488 distance was quantified; unlike the structural analyses, this correlation analysis is based on simulations in which the clamshell actually opens wide, to sample the broadest possible interval of clamshell opening. The correlation coefficient is −0.86 for the two simulations with largely open clamshell in GluK2-*trans* (simulations #4 and #5), and it is −0.89 in GluK2-*cis* (simulation #2), see Fig 5 for correlation diagrams. Such a high correlation demonstrates how the opening and closing of the LBD clamshell translates into force that controls the structure of the ion channel.

Note that the decrease of the linker distance in response to the opening of the clamshell is overestimated in our simulations of single LBD monomers, for two reasons. As discussed above, since there are no sterical clashes with the D2 domain on an interacting monomer, the clamshell is free to open too wide, leading to an overestimated shortening of the linker distance. In addition, the interface between individual monomers in a dimer exhibits a certain flexibility, as reported in Ref. [39]. The transduction of the clamshell movement was shown to be weakened, and the linker distance is expected to change less than what is predicted on the basis of rigid orientation of monomers. Since this is the case in our virtual dimer trajectories, the linker response is overestimated. A comprehensive discussion of this issue can be referred to in Ref. [16] (supporting information); aiming merely at the qualitative comparison of the isomers of *gluazo*, the virtual dimer setup is considered reasonable.

### Ligand–Protein and Intradomain Interactions in GluK2-*trans* and GluK2-*cis*

**Fig 6:**
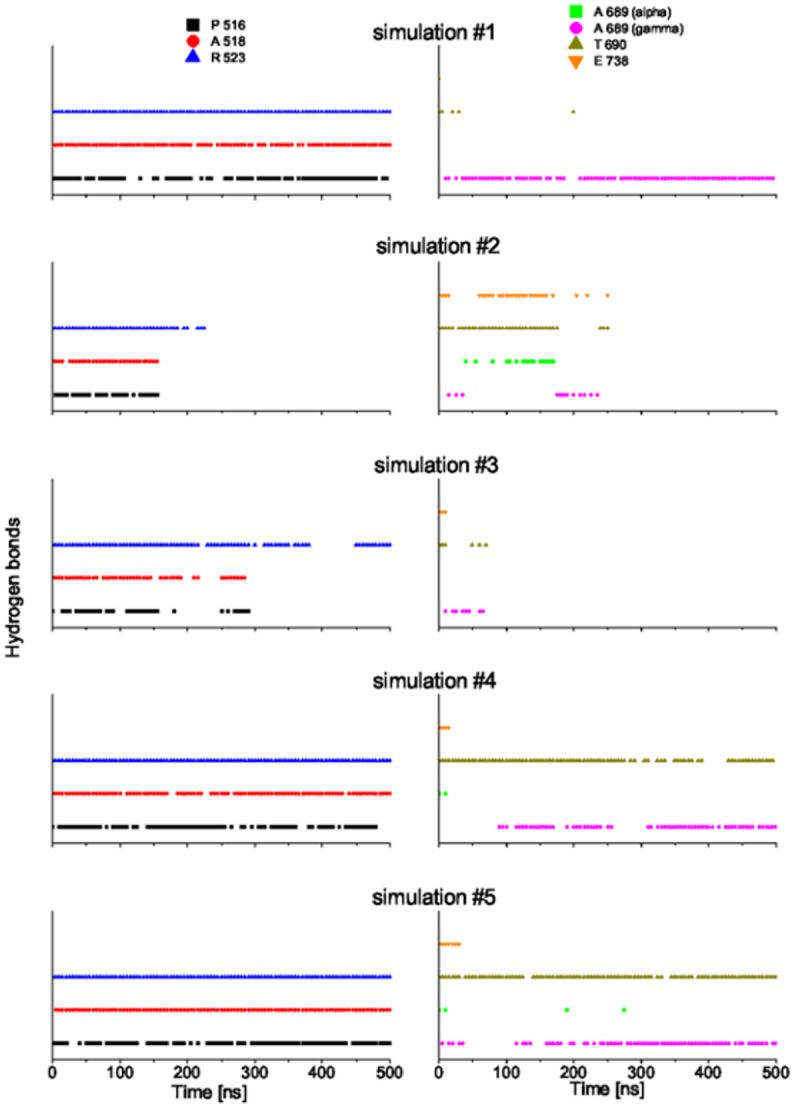
Hydrogen bond patterns in the GluK2-*cis* complex in course of MD simulations of 500 ns. See the caption of Fig 3 for the legend.

**Fig 7:**
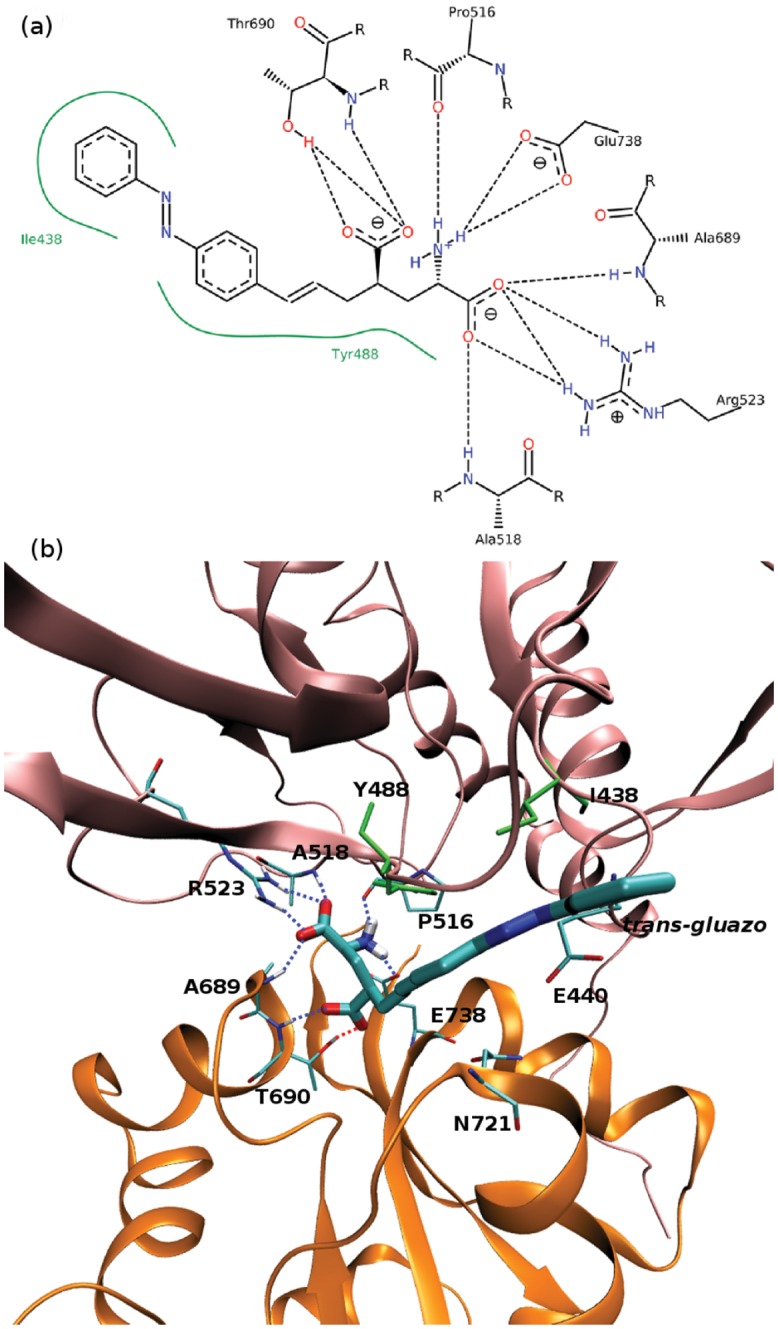
Ligand–protein interactions in the GluK2-*trans* complex. (a) 2D diagram of the ligand–protein interactions generated by PoseView.[45–47] Blue and red dashed lines—hydrogen bonds, green dashed lines—*π*-cation interactions, green lines—hydrophobic interactions. Default parameters were used to identify the interaction patterns, as presented in the manual at http://www.biosolveit.de/poseview. (b) 3D diagram of the ligand–protein interactions. Tubes colored by atom—ligand and contacting residues within the receptor, cartoon mode—receptor, pink—LBD D1 domain, orange—D2 domain, green—residues engaged in hydrophobic interactions with the ligand, dotted lines—hydrogen bonds.

**Fig 8:**
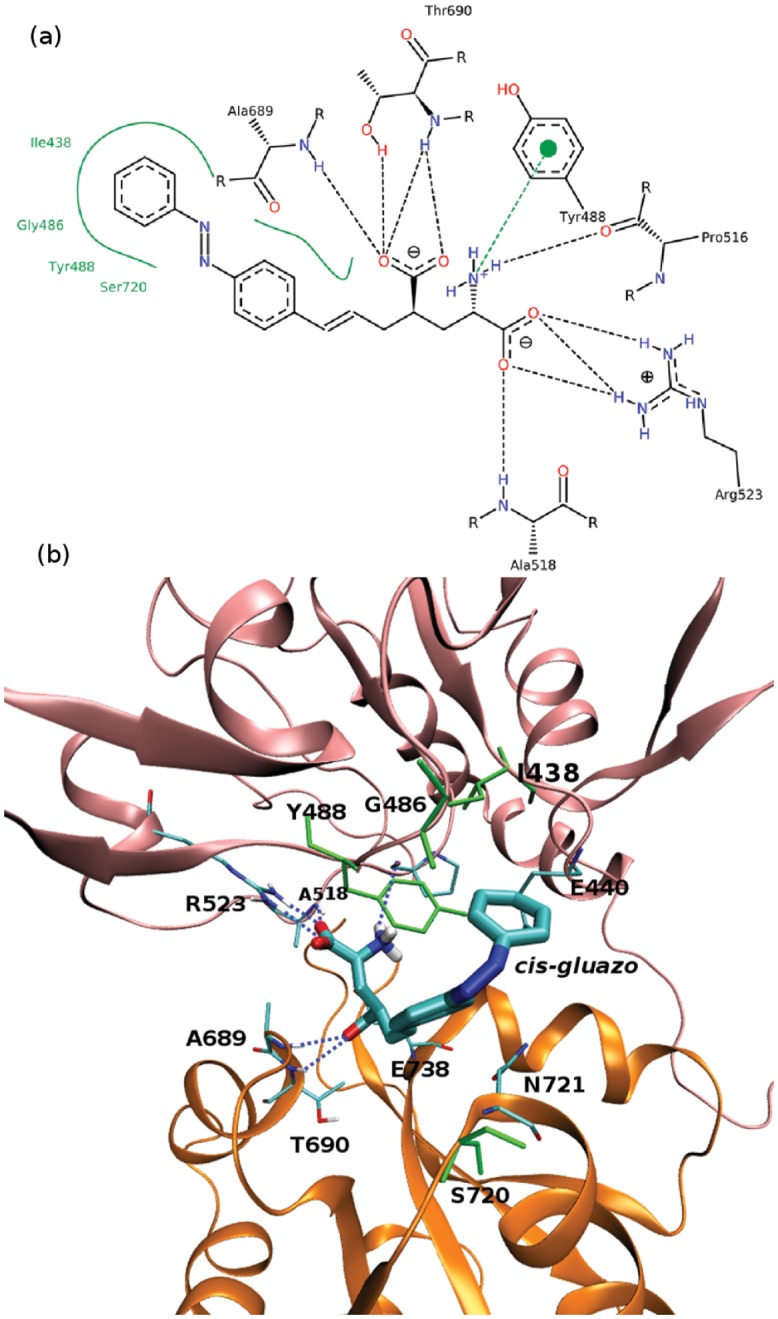
Ligand–protein interactions in the GluK2-*cis* complex. (a) 2D diagram of the ligand–protein interactions. See the caption to Fig 7a for legend. Note that the used software cannot distinguish between the different configurations of *gluazo*, and so *cis-gluazo* is displayed in the *trans* configuration. (b) 3D diagram of the ligand–protein interactions. See the caption to Fig 7b for legend.

The time evolution of hydrogen bond patterns between *gluazo* and the receptor is presented in Figs 5 and 6 for five simulations of both GluK2-*trans* and GluK2-*cis* complexes, and the corresponding interactions are visualized in Figs 7 and 8. For comparison, the interactions in the GluK2-*glutamate* complex may be referred to in S4 Fig. The glutamate moiety of *trans-gluazo* mainly forms HBs and salt bridges with residues P516, A518, R523 in D1 domain and residues A689, T690, E738 in D2 domain (simulations #1, #3). The ligand-D1 domain interactions, i.e. the HB between the *α*-amino acid group of *gluazo* and the backbone carbonyl group of P516, the HB between the *α*-carboxyl group of *gluazo* and the backbone NH group of A518, as well as the salt bridge with the guanidinium group of R523 are conserved in GluK2-*trans* and GluK2-*cis* complexes. They are unaffected by the isomerization of *gluazo*, implying that they may be the anchor interactions maintaining the GluK2-*gluazo* complex. Note that a previous experimental study proved R523 to be conserved in all of the glutamate receptors and to serve as the primary anchor for the *α*-carboxyl group of ligands bound to GluA2 [40].

In terms of the ligand–D2 interactions, the two complexes show some differences. The HB acceptor for the backbone NH group of A689 is preferred to be the *α*-carboxyl of *trans-gluazo*, while it is preferred to be the *γ*-carboxyl of *cis-gluazo*. Simulation #1 of GluK2-*trans* samples the HBs (A689)NH⋯*α*-carboxyl(*trans-gluazo*) and (A689)NH⋯*γ*-carboxyl(*trans-gluazo*) nearly equally, while mainly the (A689)NH⋯*α*-carboxyl(*trans-gluazo*) HB occurs in simulation #3. As for GluK2-*cis*, all the three simulations #1, #4, #5 are dominated by the (A689)NH⋯*γ*-carboxyl(*cis-gluazo*) HB on the 500 ns simulation time scale. This is of importance because the ligand–A689 interaction was proved to be a crucial determinant for the receptor desensitization rate, which correlates with the clamshell closing degree in GluK2 directly [41].

As illustrated above, the *α*-carboxyl of both *trans-gluazo* and *cis-gluazo* binds with P516, A518 and R523 in D1 domain. Thus, the observed interaction of the *γ*-carboxyl of *cis-gluazo* with A689, which is located on helix F in D2, indicates that this helix has to have shifted downwards notably. This change of the ligand–A689 interaction pattern corroborates the limited clamshell closing in GluK2-*cis*. Further, the HB between the *α*-amino group of *gluazo* and the E738 side chain, which occurs in the *trans-gluazo*-bound state, is almost lost upon isomerization. The importance of the corresponding HB in GluA4 was indicated in an earlier experimental study, in which ^3^H-AMPA does not bind to GluA4 after E706 (corresponding to E738 of GluK2) was mutated to Q706 [42]. And later, it was crystallographically detected to be involved in LBD clamshell closure upon ligand binding [37]. Moreover, the previous spectroscopy study of GluA2 shows that the interactions between the *α*-amino group of the ligand and the protein plays a large role in controlling the extent of receptor activation [43]. We infer that the disapperance of the *cis-gluazo*-NH_3_–E738 HB is one of the reasons for the clamshell opening wider in the *cis-gluazo*-GluK2 complex.

Except for the ligand-domain interaction, the D1–D2 interdomain interaction is also crucial for the opening and closing motions of the LBD clamshell. The HBs between E440 (on D1) and N721 (on D2) may be interpreted as a proxy for the D1–D2 interdomain interaction. The occurrence of the E440–N721 HB in the complexes GluK2-*trans* and GluK2-*cis* is presented in Table 1 in the form of percentages of the simulation time; the GluK2-*glutamate* complex is included as a reference. In the simulations that sample the closed state of LBD (GluK2-*glutamate*), the HB E440–N721 is present in ca. 80% of the simulation time. The simulations #1 and #3 of GluK2-*trans*, in which the clamshell closure degree is intermediate between the closed state and the open state, show slightly lower occurrences of the E440–N721 HB than the GluK2-*glutamate* complex does. The simulations #1, #4 and #5 of GluK2-*cis* show rather low occurrence of the E440–N721 HB. Therefore, the interdomain interactions are rather hindered by *cis-gluazo*, when compared to *trans-gluazo* or even glutamate. This obstruction destabilizes the closed conformation of the LBD clamshell [41].

**Table 1:** The occurrence of the E440–N721 hydrogen bond in the five independent simulations of 500 ns for each of the complexes GluK2-*trans*, GluK2-*cis* and GluK2-*glutamate*, expressed as the percentage of the total simulation time.

	GluK2-*trans*	GluK2-*cis*	GluK2-*glutamate*
1	76%	15%	82%
2	40%	21%	90%
3	65%	4%	72%
4	5%	47%	83%
5	19%	12%	74%

### Umbrella Sampling Simulations

**Fig 9:**
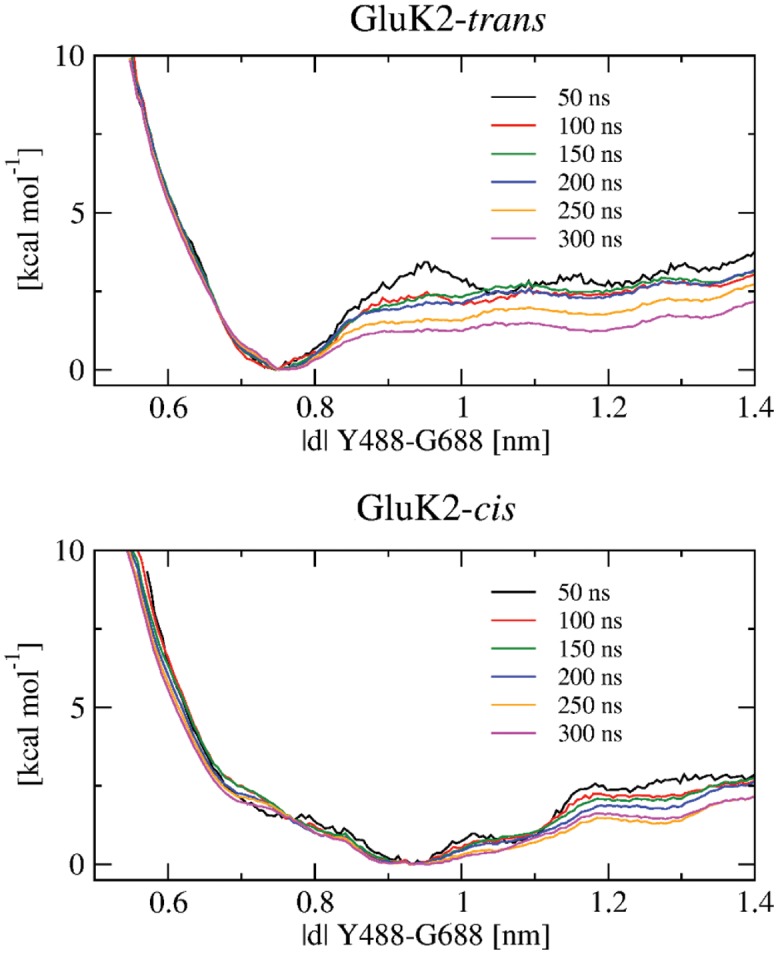
Free energy profiles for the clamshell opening/closing of the complexes GluK2-*trans* (top) and GluK2-*cis* (bottom). Each of the different curves was obtained from a series of umbrella sampling simulations of the length specified in the legend. Each free energy curve is shifted such that its value in the respective global minimum vanishes.

The conformational changes of GluK2-*trans* and GluK2-*cis* complexes were investigated by means of umbrella sampling simulation, to alleviate the issue of insufficient sampling in free MD simulations, similarly to our previous studies [16, 18]. The structure resulting from the extended equilibration procedure was used as a starting point for the umbrella sampling simulations, for each of the two complexes. These two starting structures possess a similar protein-ligand interaction pattern. The reaction coordinate was defined as the COM distance between Y488 and G688, and an interval from 0.5 nm to 1.4 nm employing 19 or 21 windows (for GluK2-*trans* and GluK2-*cis*, respectively) was spanned to fully describe the clamshell motion. The initial structure for US simulations in every window was created with a pulling simulation, in which the Y488–G688 distance was increased gradually, with a rather slow rate of 10^−5^ nm⋅ps^−1^ to avoid distortion of the protein structure. A harmonic biasing potential with a force constant of 500 kcal⋅mol^−1^⋅nm^−2^ was applied in each US window, and the amount of sampling in each window was 300 ns. The quality of sampling and convergence of simulations was assessed by the overlap of histograms (see S5 Fig) and evaluation of free energies from simulations with gradually increasing length (see Fig 9).

The potential of mean force (PMF) of clamshell opening in GluK2-*trans* shows a minimum at 0.76 nm, corresponding to the previously obtained equilibrated structure (Fig 9). The Y488–G688 distance of 0.76 nm is shorter than in the partially open state (0.82 nm, PDB ID 2XXT, bound with kainate) but still longer than in the closed state (0.63 nm, PDB ID 1S50, bound with glutamate), most likely owing to the bulky azobenzene moiety. Besides, the PMF exhibits a small barrier of ca. 1.5 kcal⋅mol^−1^ to reach the open state. This implies *trans-gluazo* to be a partial agonist, which is in agreement with the experimental study by Reiter et al. [17]. Correspondingly, the opening of the clamshell was observed in three out of five standard MD simulations (trajectories #2, #4 and #5; red, blue and orange in Fig 4 left). Especially, the simulation #2 (red) shows the structure corresponding to the open state minimum at 1.20 nm. The energy curve is flat between 1.20 and 1.35 nm, and so there is almost no restriction to open the clamshell wide. Note that the wide-open state is likely over-stabilized due to our simulation setup featuring a monomer with no interfacial restriction, which would be however present in a LBD oligomer.

In the GluK2-*cis* complex, the PMF features a free energy basin with a minimum around 0.93 nm, again corresponding to the previously obtained equilibrated structure. This minimum is separated from the open state at 1.28 nm by a barrier of 1.6 kcal⋅mol^−1^ (Fig 9). In agreement with this low barrier, opening of the clamshell was also observed in one of the free MD simulations (#2, red in Fig 4 right). Based on our simulation, the free energy barrier to open the clamshell is comparable for GluK2-*trans* (1.5 kcal⋅mol^−1^) and GluK2-*cis* (1.6 kcal⋅mol^−1^).

It is worth mentioning that it is necessary to break three HBs and one salt bridge in order to open the clamshell, judging by the structure of our starting molecular model. Taking this into account, the obtained free energy barrier of ca. 1.5 kcal⋅mol^−1^ appears rather small. However, rather than a simple breaking of the interactions between the ligand and the D2 domain, the opening of the clamshell is a more complex process that involves a formation of alternative interactions as well. For illustration, we may notice how the interactions in GluK2-*trans* are changing during the initially performed pulling simulation. The HB between the backbone NH group of A689 and *gluazo* switches from *α*-COO to *γ*-COO of *gluazo* (at 19–24 ns), and it remains at *γ*-COO until this HB breaks at 40 ns, simultaneously with the HB between T690-NH and *γ*-COO of *gluazo*. The salt bridge between the amino group of *gluazo* and E738-COO breaks and reforms at 21–24 ns, and it breaks altogether later. Finally, the HB between the hydroxyl group of T690 and *γ*-COO of *gluazo* breaks at 41 ns, after which the clamshell reaches the open state.

## Discussion and Conclusion

The mechanism of response of the GluK2 receptor to the isomerization of *gluazo* is investigated in the present study, motivated partly by the fact that the structure of the *cis-gluazo* bound GluK2 complex has not been resolved yet.

The conformation of GluK2-*cis* for *cis-gluazo*-bound GluK2 was obtained with the application of a forced switching protocol. (Technically, the complete photo-reaction could be simulated with an appropriate QM/MM model, however this is unnecessary in this study.) Our simulations indicate that both *trans-gluazo* and *cis-gluazo* bind to the LBD through anchor points represented by the backbone carbonyl of P516, the backbone amide of A518 and the guanidinium group of R523 in the D1 domain. The degree of closing of the LBD may be quantified as

$$\begin{array}{c} \text{CD}=\frac{{\left|d\right|}_{\text{open}}-\left|d\right|}{{\left|d\right|}_{\text{open}}-{\left|d\right|}_{\text{closed}}} \end{array}$$ (1)

where ∣*d*∣ is the Y488–G688 distance for which CD is being calculated, ∣*d*∣_open_ = 1.15 nm for the antagonist bound GluK1 (PDB ID 2QS1), and ∣*d*∣_closed_ = 0.63 nm for the agonist-bound state (with glutamate, PDB ID 1S50). The average CD is 62% for GluK2-*trans* (from two simulations, #1 and #3), 40% for GluK2-*cis* (from three simulations, #1, #4 and #5), and it is 50% in a previously studied GluK2-partial agonist complex (PDB ID 2XXT). The free energy barrier for the opening of the clamshell in both GluK2-*trans* and GluK2-*cis* complexes is rather low. Thus, *gluazo* is partial agonist in both *trans* and *cis* configurations. Mayer et al. suggested that the degree of closing of the clamshell reflects the affinity of ligand to GluK2 [10], according to which *cis-gluazo* exhibits a lower affinity than *trans-gluazo*, which is in accordance with the published dose-response curves [44].

The fundamental reason for distinct ligand affinity is the difference in the ligand–receptor and intradomain interactions in the binding pocket. We have identified three amino acids in the D2 domain that control these interactions. The contacts of the ligand with the residues A689 and E738 are beneficial for maintaining the closed state of the clamshell. The interaction of N721 with E440 facilitates the ligand affinity as reported in the study of the GluK2-kainate and GluK2-domoate complexes [41]. However, the spatially extended azobenzene moiety of *gluazo* collides sterically with N721, blocking the D1–D2 contact via N721.

The protein exhibits no ultra-fast structural transition, rather, the local structure accommodates to the isomerization product, and a real protein response follows on a 100ns time scale. The ligand-gated activation of the receptor consists of two processes: (i) the binding of the ligand to the LBD, and (ii) the conformational transition of the LBD [40]. The noticeable correlation between the opening of the binding pocket and a conformational transition of the transmembrane domain confirms the opening of the channel as a consequence of the photo-isomerization of *gluazo*.

The current MD simulation study contributes to the understanding of the response of the protein receptor to the photo-isomerization of *gluazo*. The interaction between *gluazo* and the D2 domain of the LBD plays a key role in the gating of ion channel activity, by way of the opening and closing of the LBD clamshell. *trans-gluazo* is a partial agonist to GluK2, while *cis-gluazo* exhibits a lower affinity. This is due to the weaker interaction of the ligand and the D1 domain with the D2 domain.

## Supporting Information

S1 Text Simulated annealing and umbralla sampling simulations. (PDF)

S1 Fig Crystal structure of GluK2 bound with *trans-gluazo*. Cartoon mode—receptor, red ball—Y488, blue ball—G688, tubes colored by atom—ligand. The main helices of the protein are labeled.
(EPS)

S2 Fig Variation of the CNNC dihedral angle during the isomerization of *trans-gluazo*. Data from five independent MD simulations; the additional restraining potential is placed on the dihedral angle at *t* = 0 fs.
(EPS)

S3 Fig RMSD of the protein backbone atoms relative to the starting structure in the course of the MD simulation of the isomerization of *trans-gluazo* in the GluK2-*trans* complex. (EPS)

S4 Fig Ligand–protein interactions in the GluK2-*glutamate* complex. (a) 2D diagram of the ligand–protein interactions. See the caption to Fig 7a for legend. (b) 3D diagram of the ligand–protein interactions. See the caption to Fig 7b for legend.
(EPS)

S5 Fig Histograms from the umbrella sampling simulations of the opening/closing of the clamshell. Left: GluK2-*trans* complex; right—GluK2-*cis* complex.
(TIFF)

S6 Fig Two ligand binding modes of *gluazo*. (a) Position 1: *gluazo* is in front of the EN gate. (b) Position 2: *gluazo* is behind the EN gate. Red dotted lines—EN gate, i.e. the hydrogen bond between E440 and N721. (E440 and N721 are located close to each other, in domain 1 and domain 2 of LBD, respectively.)
(EPS)

S7 Fig Histograms from the umbrella sampling simulations of the transfering of *gluazo* from position 1 to position 2. Left—GluK2-*trans* complex; right—GluK2-*cis* complex.
(TIFF)

S8 Fig Free energy profiles for the transition of *gluazo* from position 1 to position 2 obtained from umbrella sampling simulations. Top—GluK2-*trans* complex; bottom—GluK2-*cis* complex. Each of the different curves was obtained from a series of umbrella sampling simulations of the length specified in the legend. Each free energy curve is shifted such that its value in the respective global minimum vanishes.
(EPS)
