# Supplementary material for: Molecular Dynamics Investigation of gluazo, a Photo-Switchable Ligand for the Glutamate Receptor GluK2
Source: PLoS One. 2015 Aug 26;10(8):e0135399. doi: 10.1371/journal.pone.0135399 (PMC4550381; doi:10.1371/journal.pone.0135399)
Supplement: S1 Text — (PDF) [file pone.0135399.s001.pdf]

Supporting Information

Molecular Dynamics Investigation of *gluazo*, a Photo-Switchable Ligand for the Glutamate Receptor GluK2

Yanan Guo[1], Tino Wolter[1], Tomáš Kubař[1], Martin Sumser[2], Dirk Trauner[2], Marcus Elstner[1*]

1 Department of Theoretical Chemical Biology, Institute of Physical Chemistry, Karlsruhe Institute of Technology, Kaiserstr. 12, 76131, Karlsruhe, Germany

2 Department of Chemistry, Ludwig-Maximilians-Universität München and Center of Integrated Protein Science, Butenandtstr. 5–13, 81377 Munich, Germany

∗ E-mail: marcus.elstner@kit.edu (ME)

Simulated Annealing Simulation

Using this technique, firstly the system can be heated up from a low temperature at which the properties of the system is interested in to a certain high temperature, and then cooled down back to the previous low temperature. During cooling, the studied system is allowed to randomly explore the conformational space. Simulated annealing is efficient in studying protein conformational fluctuations, which has been successfully used by some researchers. Cordes et al. [1] studied the structure of Vpu which is a HIV-1 auxiliary protein through simulated annealing simulation, and they found five conformations for the homo-pentameric Vpu bundles. In Mayewski's research [2], the authors utilized simulated annealing to search for the global minimum of tested proteins successfully. In the present study, we use this annealing technique to search for the possible conformation besides the X-ray structure. The structure after geometry optimization was used as starting structure for simulated annealing simulation. For the data collection period, the complex was directly cooled down from 450 K, 445 K, 440 K, 435 K, 430 K, respectively, to 300 K in the first 100 ns, and then was kept at 300 K for another 100 ns.

The X-ray structure resolves the conformation that *trans-gluazo* is in front of the "E440-N721 gate" (referred as EN-gate next, S6 Fig., a). The residues E440 and N721 locate close to each other, opposite at D1 domain and D2 domain, respectively. The hydrogen bond (HBond) between E440 and N721 contributes to this EN-gate. During the high temperature simulation, *trans*-azobenzene relocated to a new position crossing the EN-gate. We define the ligand position in front of the EN-gate as postion 1 (P 1), and that behind the EN-gate as position 2 (P 2) (S6 Fig., viewed from the mouth side of the clamshell). Then

we have two complexes bound with *trans* at P 1 and P 2, and they are defined as GluK2-*trans*-P1 and GluK2-*trans*-P2.

Umbrella Sampling Simulation

We performed umbrella sampling simulation with GluK2-*trans*-P1 complex, to study the free energy properties of these two ligand binding modes for *trans-gluazo*. The reaction coordinate was defined as the COM distance between the azobenzene group and a group of receptor amino acid residues to generate a distance ensuring that the ligand is moved from P 1 to P 2. We sampled the reaction coordinate from 1.75 nm to 0.80 nm in order to fully describe the complete conformation change of the complex. A very small pulling speed of 10^{-5} nm·ps^{-1} was used to avoid unphysical distortions within the protein structure. We got 21 US windows spaced by an equidistance of 0.05 nm, with a biasing force constant of ca. 500 kcal·mol^{-1} ·nm^{-2}. A 300 ns length MD simulation was performed for each window. The quality of sampling and convergence of simulations was assessed by the overlap of histograms (see S7 Fig., left) and evaluation of free energies from simulations with gradually increasing length (see S8 Fig.). The PMF changes slightly after 200 ns, indicating the convergence of the simulation. The PMF curve after 300 ns shows an energy barrier of ca. 14.4 kcal·mol^{-1} from P 1 to P 2, indicating that the binding mode for *trans-gluazo* at P 1 is more stable than that at P 2.

We performed the same study for *cis-gluazo*. Firstly we isomerized GluK2-*trans*-P1 and GluK2-*trans*-P2 to GluK2-*cis*-P1 and GluK2-*cis*-P2, respectively. Then a similar umbrella sampling simulation for transferring the *cis-gluazo* from P 1 to P 2 was performed with the GluK2-*cis*-P1 complex as for GluK2-*trans*-P1 above, but with the reaction coordinate defined from 2.20 nm to 1.20 nm between the COM of benzene group and receptor residues different from GluK2-*trans*-P1. This is because azobenzene group has different orientation in the two complexes. A convergence is depicted by a good histogram overlap of each window shown in (S7 Fig., right) and evaluation of free energies from simulations with gradually increasing length in (S8 Fig.). The PMF suggests that *cis-gluazo* prefers staying at P 1 then at P 2 with the energy barrier of ca. 3.47 kcal·mol^{-1}.

References

1. Cordes FS, Kukol A, Forrest LR, Arkin IT, Sansom MSP, et al. The structure of the HIV-1 Vpu ion channel: modelling and simulation studies. Biochimica et Biophysica Acta (BBA) - Biomembranes 2001;1512: 291–298.

2. Mayewski S A multibody, whole-residue potential for protein structures, with testing by Monte Carlo simulated annealing. Proteins: Structure, Function, and Bioinformatics 2005;59: 152–169.
