# Supplementary figures and images for: Molecular Dynamics Investigation of gluazo, a Photo-Switchable Ligand for the Glutamate Receptor GluK2
Source: PLoS One. 2015 Aug 26;10(8):e0135399. doi: 10.1371/journal.pone.0135399 (PMC4550381; doi:10.1371/journal.pone.0135399)

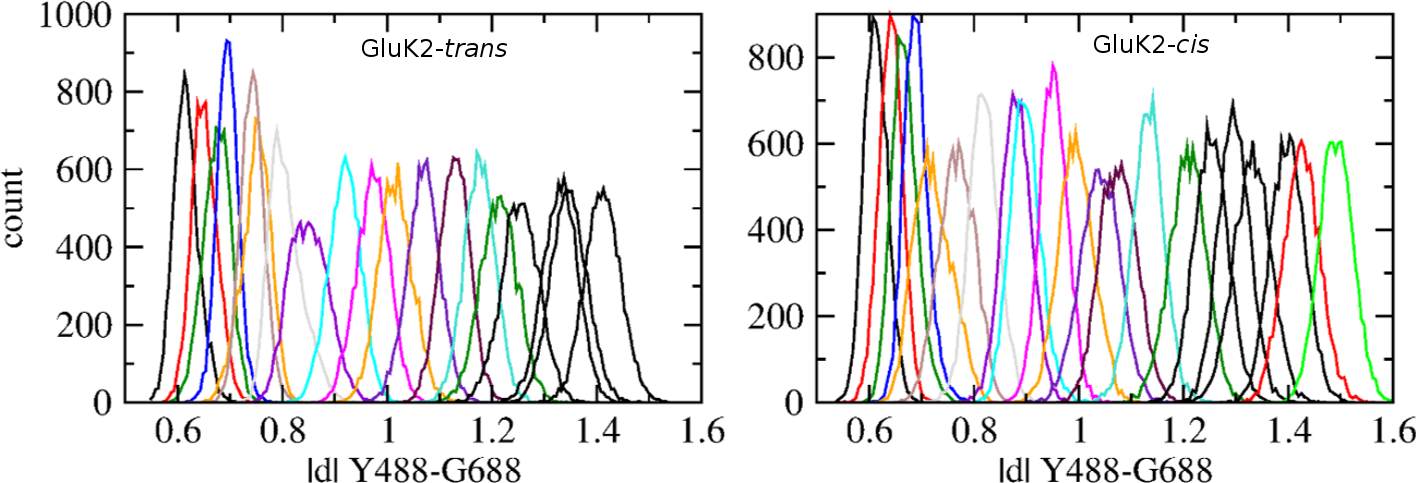

Supplement: S5 Fig — Left: GluK2-trans complex; right—GluK2-cis complex. (TIFF) [file pone.0135399.s006.tiff]

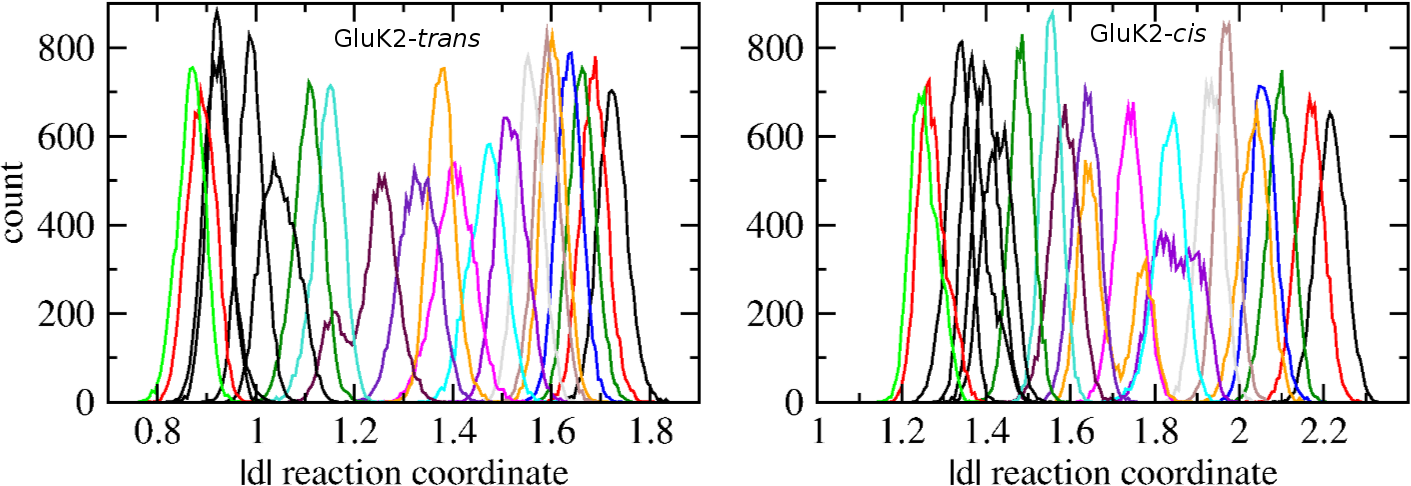

Supplement: S7 Fig — Left—GluK2-trans complex; right—GluK2-cis complex. (TIFF) [file pone.0135399.s008.tiff]
